# Research and Experiments on an External Miniaturized VFTO Measurement System

**DOI:** 10.3390/s20010244

**Published:** 2019-12-31

**Authors:** Lizhe Wang, Wenbin Zhang, Xiangyu Tan, Weiren Chen, Shiqi Liang, Chunguang Suo

**Affiliations:** 1 Faculty of Science, Kunming University of Science and Technology, Kunming 650504, China; wanglizhe@stu.kust.edu.cn (L.W.); suochunguang@kust.edu.cn (C.S.); 2 Faculty of Mechanical and Electrical Engineering, Kunming University of Science and Technology, Kunming 650504, China; cwrfjl@stu.kust.edu.cn (W.C.); liangshiqi@stu.kust.edu.cn (S.L.); 3 Electric Power Research Institute, Yunnan Power Grid Co., Ltd., Kunming 650217, China; 2040464@163.com

**Keywords:** very fast transient overvoltage (VFTO), measurement system, differentiating–integrating system, capacitive voltage divider, field measurement

## Abstract

Disconnect switch and circuit breakers operations in gas insulated switchgear (GIS) systems may produce very fast transient overvoltage (VFTO). Detecting VFTO is the first step for researchers to reduce the damage to other equipment of the substation caused by VFTO. Most of the existing sensors used for VFTO are generally bulky, complex to install, and require modification of the GIS structure. In this paper, a miniaturized measurement system that uses capacitive voltage divider and differentiating–integrating circuit is proposed. A special sensor structure and optimized differentiating–integrating circuit components arrangement were designed to increase the bandwidth of the measurement system. The frequency-domain, time-domain and voltage divide calibration experiment was performed, and a comparison experiment with an internal VFTO sensor was conducted. The measurement system was applied in the 500 kV GIS substation, and the VFTO measurement under specific conditions was carried out. The measured time domain and frequency domain waveforms conformed to the definition of standard VFTO according to IEC 60,071. It was found that the proposed measurement system meets VFTO measurement requirements and can be applied to actual VFTO measurements.

## 1. Introduction

Gas-insulated substation (GIS), an equipment filling with SF_6_, has been widely used in power systems since the 1960s. Compared with the traditional air-insulated substation (AIS), it has the advantages of high safety factor, less space, long maintenance period and good arc-extinguishing performance. However, since the circuit breaker and the isolating switch operate at high voltage, voltage pre-breakdown and restrike at the contact will generated, and electromagnetic wave will produce refraction at the discontinuity of the wave impedance, resulting in a very fast transient overvoltage (VFTO) [1,2,3,4,5,6,7]. The VFTO wave head is steep, the amplitude reaches up to 2.7 p.u. [8] and the rise time of the waveform is several ns to ten ns [9]. According to IEC60071-1, the frequency component of VFTO is composed of three parts: (1) in the GIS HV busbar, a very high frequency component formed by several slight changes in wave impedance, up to 100 MHz; (2) at the end of the GIS HV busbar and the end of the cable or overhead line, the high-frequency component formed by the significant change in the wave impedance causes reflections up to 30 MHz and (3) low-frequency components caused by external large-capacitance equipment resonance ranging from 0.1 to 5 MHz, such as capacitive voltage transformers or coupling capacitors of power line carrier systems. Although the duration of breakdown is extremely short under the action of SF_6_ at a certain pressure, short-time high-amplitude pulses still cause EMP interference to GIS internal equipment and external equipment. In recent years, the damage caused by VFTO to the power system has aroused researcher’s attention [10,11].

As a result, in order to capture the VFTO signal accurately and provide the measured data for the subsequent elimination of VFTO, the embedded electrode method [12,13], the basin insulator wraparound electrode differentiating–integrating measurement method [14], the GIS hand hole method [15,16,17,18,19,20,21,22,23], etc. have been developed, and all obtained certain measured data. For the embedded electrode method, although the frequency band is wide, it is necessary to pre-bury the measuring electrode when manufacturing the GIS. After the GIS is put into operation, this method cannot be used to measure the VFTO. For the basin insulator wraparound electrode differentiating–integrating measurement method, although the GIS that is put into operation can be measured, since the installation method is wrap measuring electrode around the insulator, the value of the capacitance to the ground is large, and the influence on the measurement is affected. For the GIS hand hole method, although it has the advantages of wide frequency band, it needs to modify GIS structural that is put into operation, and the modification process is complicated, which is not allowed by the power department.

Summarize existing VFTO measurement methods, although the internal sensor can directly measure the VFTO inside the GIS tank, it needs to modify the existing GIS structural. The external sensor is complicated to install, and the parameters are affected by the installation method. The strip-shaped measuring electrode is prone to edge effects, causing electric field distortion, the time at which the distributed charge on the measuring electrode reaches the electrode lead is different, the frequency band of the measurement system is lowered, and the measured waveform is distorted. For high-voltage GIS equipment, an external measurement system is required for both its performance and compact size. In the research for this paper, a measurement system comprising a capacitive voltage divider and a differentiating–integrating circuit was designed, fabricated and tested. The design of the measurement system was based on optimized differentiating–integrating system. Critical parameters of the measurement system, including value of electronic component in the circuit and structure of the voltage divider was designed and simulation to meet the requirement of VFTO measurement. When system was fabricated, some test was run to verify performances. Using a standard signal generator and a calibration platform, the time-domain and frequency-domain characteristic of the measurement system, which describe measuring ranges of the system, were measured and verified. A voltage division ratio calibration platform was built to test the measurement system. After the system is tested and verified, we compared it with a standard internal VFTO sensor to confirm the system meets VFTO measuring requirements, and a field measurement in 500 kV Dali Substation is done. Compared with the internal sensors, the measurement system in this paper has the advantages of small size, easy installation and fixation, and no need to modify GIS structural when measuring. Compared with external sensors, the parameters of this measurement system are not affected by the installation method, and the frequency band is similar to the internal sensor, which can accurately measure the VFTO signal.

This paper has five parts: Section 1 is the introduction; Section 2 is the design, optimization and simulation of the measurement system; Section 3 describes calibration and verify of the measurement system; Section 4 shows a field test to measuring VFTO signal using this measurement system and Section 5 presents the conclusions.

## 2. Principle and Parameter Design of the VFTO Measurement System

### 2.1. Principle of Differentiating–Integrating Measurement

**Figure 1:**
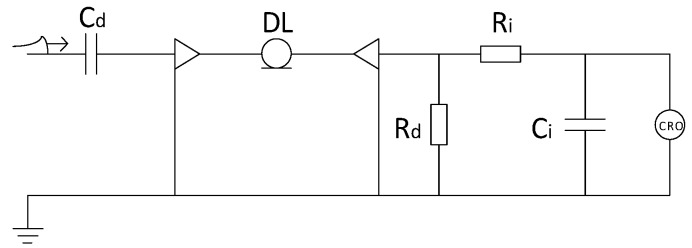
Simplified differentiating-integrating measurement system equivalent circuit.

The principle of the classic differentiating–integrating measurement system is shown in Figure 1, where $C_{i}$ and $R_{i}$ constitute a passive first-order integral unit, $C_{d}$ and $R_{d}$ constitute a differential unit, DL is the coaxial cable, the backend CRO is an oscilloscope and the internal resistance and capacitance of the oscilloscope are ignored. $R_{d}$ also serves as the matching resistor at the end of the coaxial cable, and its value is the same as characteristic impedance of coaxial cable, much smaller than the equivalent capacitive reactance formed by $C_{d}$. The differential capacitor $C_{d}$ is a high voltage component. In the conventional basin insulator wraparound electrode differentiating–integrating measurement method, $C_{d}$ is the high voltage capacitor formed by the measuring electrode and the HV busbar.

The front-end sensor is the differentiator, and the output is a differential signal, which needs to be restored by the integrator and output to the oscilloscope. The integrator can be selected using a passive integrator, an active integrator or a better performing hybrid integrator depending on the measurement accuracy requirements of the instrument used.

According to the model above, the voltage transfer function of this circuit can be described as

(1) $$G\left(s\right)=\frac{R_{d}}{R_{d}+\frac{1}{C_{d}s}}\times \frac{1}{R_{i}C_{i}s}=\frac{R_{d}C_{d}}{\left(1+R_{d}C_{d}s\right)R_{i}C_{i}},$$

where $T_{d}=R_{d}C_{d}$ is the derivative time constant and $T_{i}=R_{i}C_{i}$ is the integral time constant. Thus, voltage division ratio can be described as

(2) $$K=\underset{s\to 0}{lim}\frac{1}{G\left(s\right)}=\frac{R_{i}C_{i}}{R_{d}C_{d}}=\frac{T_{i}}{T_{d}}.$$

To ensure adequate measurement accuracy, it is required that

(3) $$\frac{1}{R_{i}C_{i}}\ll \omega \ll \frac{1}{R_{d}C_{d}},$$

where ω is the equivalent frequency of measured voltage.

The step response time of the classical differentiating–integrating measurement system can be described as

(4) $$T=-K\underset{s\to 0}{lim}\frac{1}{G^{\prime \left(s\right)}}=R_{d}C_{d}.$$

**Figure 2:**
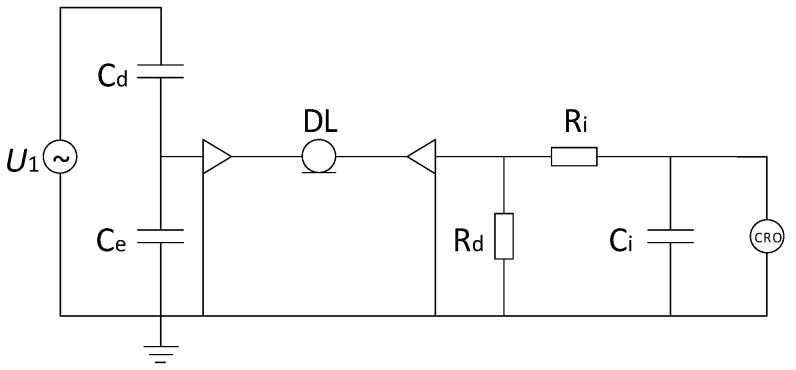
Equivalent circuit after considering the stray capacitance of the measuring electrode to ground.

In the actual measurement, capacitance and inductance of the coaxial cable cannot be ignored, and the electromagnetic environment of the substation is complex. Uncertain factors such as stray capacitance and residual inductance make the components of the integral and differential parts non-ideal, and these factors will affect the measurement system. As a result, structure and components of the differentiator and integrator need to be modified to eliminate the stability degradation caused by the high frequency characteristics of the device. Besides, the equivalent frequency of the VFTO measurement is low, whether it will take influence has yet to be verified, in order to simplify the modeling and parameter calculation, the uncertain factors is ignored. However, the above simplified model ignores the capacitance formed by the sensor’s induction plate and the shielding layer, and the stray capacitance formed by the sensor to the ground. Adding stray capacitance $C_{e}$ to the model, the equivalent circuit of the differentiating–integrating measurement system according to the actual situation is shown in Figure 2.

The transfer function of this circuit can be described as

(5) $$G\left(s\right)=\frac{R_{d}C_{d}/R_{i}C_{i}}{R_{d}\left(C_{d}+C_{e}\right)s+1}.$$

Thus, the voltage division ratio is

(6) $$K=\underset{s\to 0}{lim}\frac{1}{G\left(s\right)}=\frac{R_{i}C_{i}}{R_{d}C_{d}}=\frac{T_{i}}{T_{d}}.$$

The step response time can be described as

(7) $$T=-K\underset{s\to 0}{lim}\frac{1}{G^{\prime \left(s\right)}}=R_{d}\left(C_{d}+C_{e}\right).$$

It can be concluded from the above that the step response time of the measurement system is related to the stray capacitance caused by the differential capacitance, differential resistance and sensor structure design difference. According to the capacitance definition formula, if the sensor has a large area to the ground, the stray capacitance between the sensor housing and the ground will be affected by the height of the installed GIS, and the sensor parameters will change, resulting in measurement errors.

### 2.2. Sensor Parameter Design

According to the VFTO waveform characteristics and IEC TS 61321-1, the overall step response time of the measurement system should be within 5 ns, and the low frequency response should be lower than the power frequency. The high frequency characteristics of the measurement system are determined by the differential part, and the low frequency characteristics are determined by the integral part, which puts higher requirements on the differential and integral parts of the measurement system.

**Figure 3:**
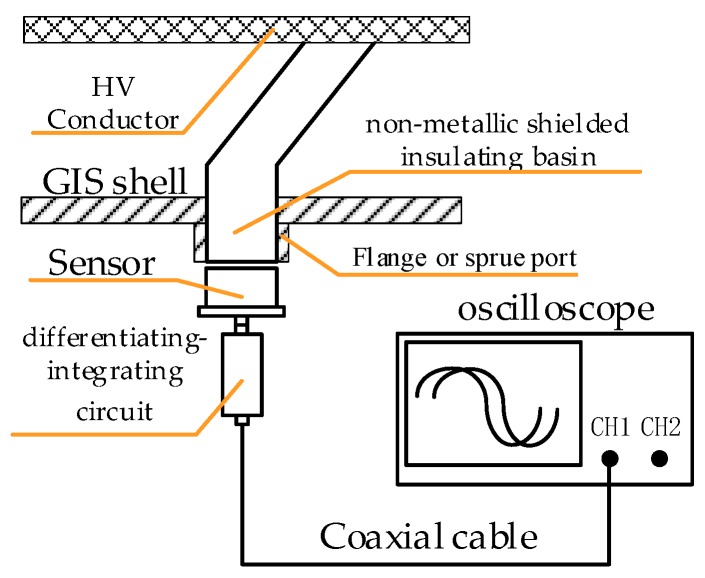
Basic structure of external very fast transient overvoltage (VFTO) measurement system.

First, the design of the differential part is performed. The basic structure of the external measurement system for measuring VFTO is shown in Figure 3. The front end is a capacitive voltage divider. Differentiating–integrating circuit is directly connected to the sensor and connected to the oscilloscope through a coaxial cable with a 50 Ω impedance. Set the oscilloscope to 50 Ω to match the matching resistor in the calculus circuit to eliminate the buckling reflection in the cable when transmitting transient signals.

We have come to the conclusion that the sensor of the measuring system needs to have two cavities to form $C_{d}$ and $C_{e}$, whether the sensor of the measuring system is installed on the non-metallic shielded insulating basin, the sprue gate or the observation window, the capacitance of the ground stray capacitance relative to the capacitance of the sensor cavity must be ignored, which requires reducing the projected area of the sensor to the ground.

The outside of the sensor can be designed to be round or rectangular, but the round sensor is difficult to fix when installed in the field, so we chose a rounded rectangle for design. $C_{d}$ and $C_{e}$ need to be implemented through the sensor structure. The sensor is designed as a circular cavity, and it is cut into two circular cavities by using induction plates to implement $C_{d}$ and $C_{e}$. Designed as a circular shape can increase the induction area, the volume is reduced as much as possible when the cavity capacitance is fixed, and the loss is small compared to the rectangular cavity. However, because the high-frequency and low-frequency characteristics of the sensor are not exactly the same, when designing the cavity of $C_{d}$ and $C_{e}$, the internal cavity of the sensor cannot be directly divided according to the capacitance.

**Figure 4:**
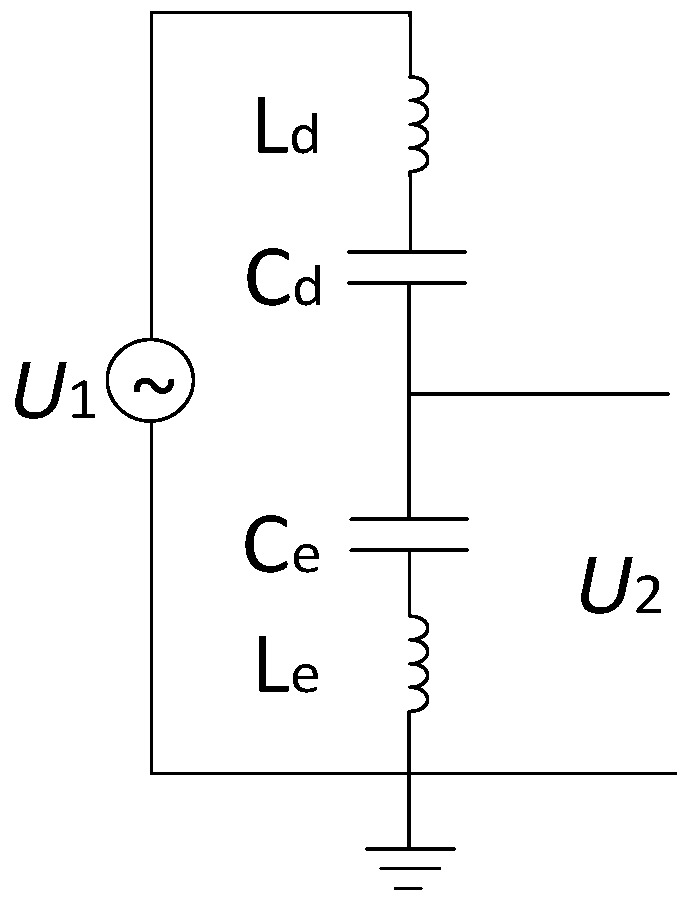
Equivalent circuit when the sensor measures high frequency signals.

When designing the high-frequency parameters of the measurement system, the capacitance sensor cannot be regarded as a simple voltage divider, and there is stray inductance affected by the sensor structure. The actual equivalent model is shown in Figure 4. We can derive the transfer function as follows

(8) $$\begin{array}{ll} H\left(s\right)=\frac{U_{2}}{U_{1}} & =\frac{I\left(2j\pi fL_{e}+\frac{1}{2j\pi fC_{e}}\right)}{I\left(2j\pi fL_{d}+\frac{1}{2j\pi fC_{d}}+2j\pi fL_{e}+\frac{1}{2j\pi fC_{e}}\right)}\\ & =\frac{-4\pi^{2}f^{2}L_{e}C_{d}C_{e}+C_{d}}{-4\pi^{2}f^{2}L_{d}C_{d}C_{e}+C_{e}-4\pi^{2}f^{2}L_{e}C_{d}C_{e}+C_{d}}. \end{array}$$

The resonant frequency of the branch to the differential capacitor $f_{d}$ and the branch to the ground capacitor $f_{e}$ can be described as

(9) $$f_{d}=\frac{1}{2\pi \sqrt{\frac{C_{d}C_{e}\left(L_{d}+L_{e}\right)}{C_{d}+C_{e}}}}.$$

(10) $$f_{e}=\frac{1}{2\pi \sqrt{L_{e}C_{e}}}.$$

From Equations (8)–(10), we have

(11) $$H\left(s\right)=\frac{U_{2}}{U_{1}}\approx \frac{\frac{f^{2}}{f_{e}{}^{2}}}{\frac{f^{2}}{f_{d}{}^{2}}}\times \frac{C_{d}}{C_{e}+C_{d}}.$$

**Figure 5:**
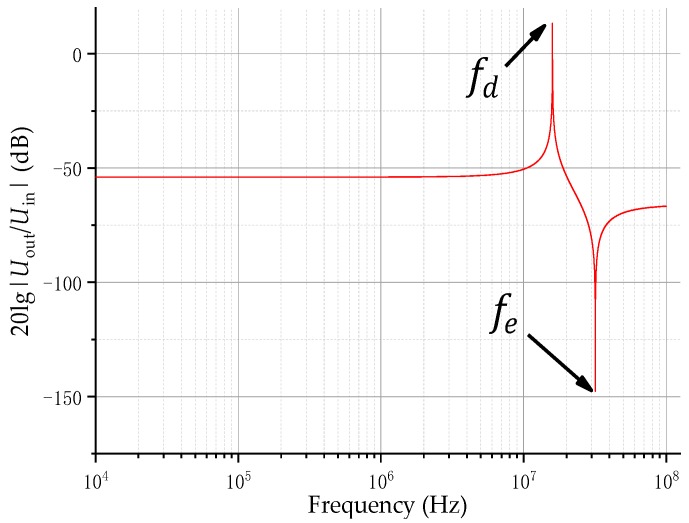
A frequency response of the capacitor divider.

In order to study the characteristics of the transfer function, assuming that $L_{d}=10\;\mu H$, $C_{d}=10\;\mathrm{pF}$, $L_{e}=5\;\mathrm{nH}$, and $C_{e}=5\;\mathrm{nF}$, and the plotted frequency response is as shown in Figure 5. In the figure, the ordinate is the transfer coefficient in decibels, and the abscissa is the frequency. The figure consists of two poles $f_{d}$ and $f_{e}$, which are the poles and zeros of the transfer function, respectively. When the measured frequency $f=f_{d}$, $C_{d}$ is connected in series with $C_{e}$, and forms a series resonant circuit with $L_{d}+L_{e}$, engendering voltage of $U_{2}$ is much larger than $U_{1}\frac{C_{d}}{C_{e}+C_{d}}$, and even higher than 0 dB under certain parameter settings, which make $U_{2}$ greater than $U_{1}$, causing transient high voltage impact on the integral part and the measuring instrument, damaging the device. When the measured frequency $f=f_{e}$, $C_{e}$ and $L_{e}$ form a series resonant circuit, the voltage of $U_{2}$ is much smaller than $U_{1}\frac{C_{d}}{C_{e}+C_{d}}$, and if it is lower than sensitivity of the measuring instrument, the signal at this frequency cannot be measured. Therefore, the factors limiting the high-frequency characteristics of the sensor are mainly the frequencies of the two resonance points, and the front edge of the first resonance point is relatively slow, so that the transmission characteristics in the sensor design bandwidth are flat, and a certain margin is required.

It can be seen from Equation (9) that $C_{d}$, $L_{d}$ and $L_{e}$ need to be reduced in order to make the resonance point larger than the design bandwidth while leaving a certain margin. The sensor’s induction plate is facing the GIS HV busbar. It is known from the capacitance definition that reducing $C_{d}$ can be achieved by increasing the plate distance or reducing the plate area, but since the sensor volume is small, increasing the distance or reducing the plate area will have an effect on $C_{e}$. In addition, when the measurement system is installed to the GIS, the distance from the sensor’s induction plate to the HV busbar of the GIS is variable, causing $C_{d}$ to change. Therefore, when designing $C_{d}$, it is necessary to ensure the high-frequency characteristics and the voltage division ratio of the sensor when measuring GIS equipment of any voltage level. According to the step response time of the measurement system of Equation (7) and the voltage division ratio formula of Equation (12), we concluded that the design of $C_{e}$ and $C_{d}$ should meet the following two points: First, the value of $C_{e}+C_{d}$ should remain the same or change as small as possible, not affected by installation methods or GIS equipment with different voltage levels; second, the value of $\frac{C_{d}}{C_{e}+C_{d}}$ should be as small as possible when measuring GIS equipment of different voltage levels.

Therefore, $C_{e}$ needs to be larger than $C_{d}$ to meet the above two requirements, and the limit condition needs to be used when designing $C_{d}$. Considering the $C_{d}$ formed by the cavity of the sensor itself when the HV busbar was attached to the sensor, the distance between the HV busbar and the sensor’s induction plate was the smallest, and value of $C_{d}$ was the largest. In any case, $C_{d}$ was less than the limit case, and the high frequency characteristics were better than the limit case.

$L_{d}$ is the stray inductance between the GIS HV busbar and the sensor’s induction plate at high frequency. The electromagnetic wave radiated outward from the VFTO could not be quantitatively analyzed, and the generated magnetic field was not a uniform magnetic field. Therefore, the quantitative calculation of $L_{d}$ was difficult, only structural design could be used to reduce mutual inductance at high frequencies. Reducing $L_{d}$ also requires increasing the plate distance or reducing the plate area. In addition to considering the effect of increasing the distance between the plates on $C_{e}$, it is also necessary to consider the problem of reducing the sensitivity of the sensor due to the reduction of the plate area.

In summary, $C_{d}$ and $L_{d}$ should be reduced without changing the sensitivity of the sensor. The cavity of the induction plate should be deeper without changing the size.

**Figure 6:**
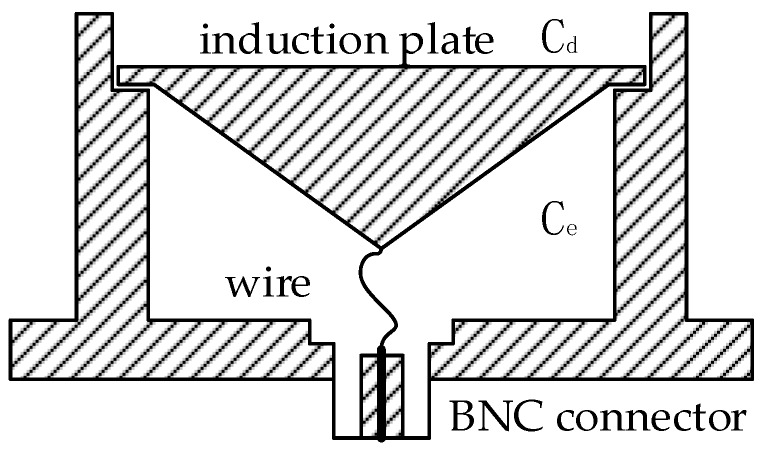
Tapered structure that reduce $L_{e}$.

$L_{e}$ is the sum of stray inductance generated under the sensor plate and the sensor case at high frequencies and stray inductance of plate lead and sensor housing. Like $L_{d}$, $L_{e}$ is difficult to calculate quantitatively. Reducing $L_{e}$ can be divided into two parts: first, reduce the stray inductance of the lower cavity at high frequencies and second, reduce the stray inductance of plate lead and sensor housing. According to the mutual inductance formula, reducing the inductance of the lower cavity at high frequencies can be achieved by reducing the area directly opposite the charged conductor. If the plate is cylindrical, the upper and lower ends are flat, and the area between the lower end and the sensor case is the smallest, but this will make the lead longer and increase the inductance caused by the lead, so it is necessary to find the balance between the two to reduce $L_{e}$. This paper used a tapered structure at the lower end of the sensor plate, which shortened the lead wire while ensuring the capacitance of $C_{e}$. Meanwhile, the area of the lower end of the electrode plate facing the sensor housing only increased slightly to minimize $L_{e}$. In addition, when the induction plate measured the signal, the induced charge was evenly distributed on the surface. If it is a flat plate structure, the time induced charge of the edge reaches the lead is longer than the time induced charge at the center of the substrate reaches the lead at high frequency due to the skin effect, the combination of the two will cause waveform distortion and adversely affect the amplitude range. The tapered structure can suppress the occurrence of redistribution, so that the difference in arrival time between the two is reduced [23]. Sectional view of the sensor is shown in Figure 6.

When designing the low frequency part of the sensor, there is no need to consider the effects of stray inductance. Therefore, the branch where the sensors $C_{d}$ and $C_{e}$ are located will not resonate, and there will be no poles $f_{d}$ and zero point $f_{e}$ of the transmission characteristic. The voltage of $U_{2}$ is equal to $U_{1}\frac{C_{d}}{C_{e}+C_{d}}$. That is to say, the equivalent circuit of the measurement system is shown in Figure 2. It can be known from Equation (3) that the corresponding characteristics of the low frequency of the system are determined by $\frac{1}{R_{i}C_{i}}$.

### 2.3. Design of Differentiating–Integrating Circuit

It can be seen from Equations (7) and (11) that the high frequency response of the system consists of the high frequency response of the sensor itself and the high frequency response of the differentiating–integrating, both of which need to meet the high frequency requirements of the VFTO measurement.

Since the quantitative calculation of the high-frequency stray inductance of the sensor is complicated, it is difficult to calculate the rise time of the pole $f_{d}$ in Figure 5, so the high-frequency response of the differentiating–integrating system needs to leave a certain margin. VFTO requires that the step response of the measurement system is less than 5 ns, leaving a 2 ns margin, which is reasonable for 3 ns. The high frequency response of the sensor is

(12) $$R_{d}\left(C_{d}+C_{e}\right)\leq 3\;\mathrm{ns}.$$

When the high-voltage equipment such as GIS is working normally, the current frequency of the busbar is the power frequency. Since the VFTO is still superimposed by part of the power frequency when the circuit breaker and the insulation switch are operated, the bandwidth of the measurement system needs to be as low as the power frequency. From the analysis of the low frequency part of the measurement system, the low frequency response of the system is determined by the differential part of the calculus circuit. Therefore, the low frequency response of the measurement system is

(13) $$\frac{1}{R_{i}C_{i}}\leq 50\;\mathrm{Hz},$$

equals

(14) $$R_{i}C_{i}\geq 3\;\mathrm{ms}.$$

In addition, the voltage division ratio of the measurement system is determined by the combination of the sensor’s voltage division ratio and the differentiating–integrating circuit’s voltage division ratio. The voltage division ratio of the sensor is $\frac{C_{d}}{C_{e}+C_{d}}$, and the voltage division ratio of the differentiating–integrating system is $\frac{T_{i}}{T_{d}}$, then the voltage division ratio of the measurement system is

(15) $$K=\frac{C_{d}}{C_{e}+C_{d}}\times \frac{T_{i}}{T_{d}}.$$

**Figure 7:**
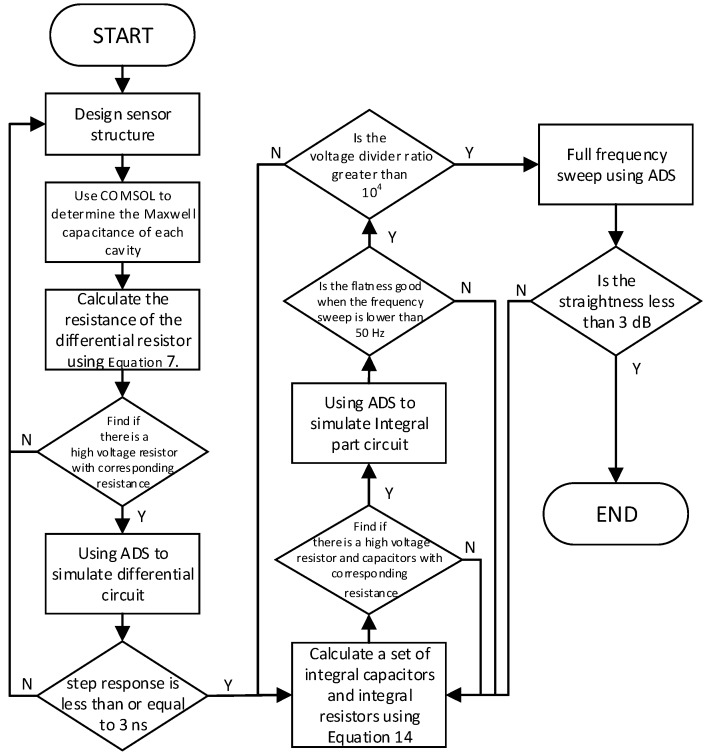
Measurement system important parameters determination process.

Under the premise of limited component resources, reasonable selection of parameters can make the measurement system reach the optimal value. Since $C_{d}$ and $C_{e}$ are determined by the structure, it is not possible to directly determine the various parameters in the circuit. After designing the structure of the sensor, we can use the COMSOL software to get the Maxwell capacitance of each cavity, also, S-parameter simulation and time domain simulation by ADS (advanced design system) can give us an optimized value of capacitance and resistance in the differentiating–integrating system. The specific process is shown in Figure 7.

**Figure 8:**
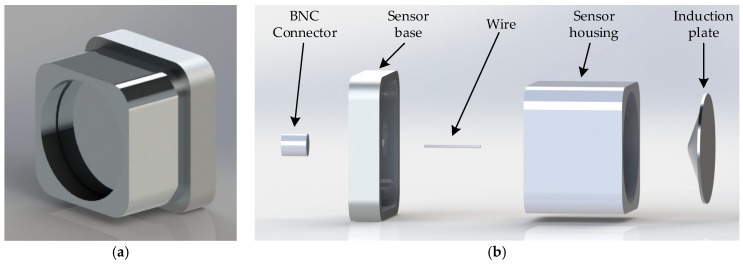
Front-end sensor rendering. (**a**) Appearance and (**b**) exploded view.

It was finally determined that $C_{d}=6\;\mathrm{pF}$, $C_{e}=20.5\;\mathrm{pF}$, $R_{d}=50\;\Omega$, $R_{i}=100\;\Omega$ and $C_{i}=0.6\;\mu F$. It could be calculated that the step response time was $1.326\;\times \;10^{-9}\;s$, low-frequency response time was $6\;\times \;10^{-5}\;s$ and voltage division ratio was larger than $10^{4}$, the appearance of the front-end sensor of the measurement system completed with parameters and structure design is shown in the Figure 8a. Figure 8b is the exploded view of the front-end sensor structure, consists of five components. The induction plate and BNC connector were connected by a wire, which were all inside sensor housing installed on sensor base. The induction plate divides the inner cavity of the sensor housing into two parts to implement $C_{d}$ and $C_{e}$, and insulated from the sensor housing.

**Figure 9:**
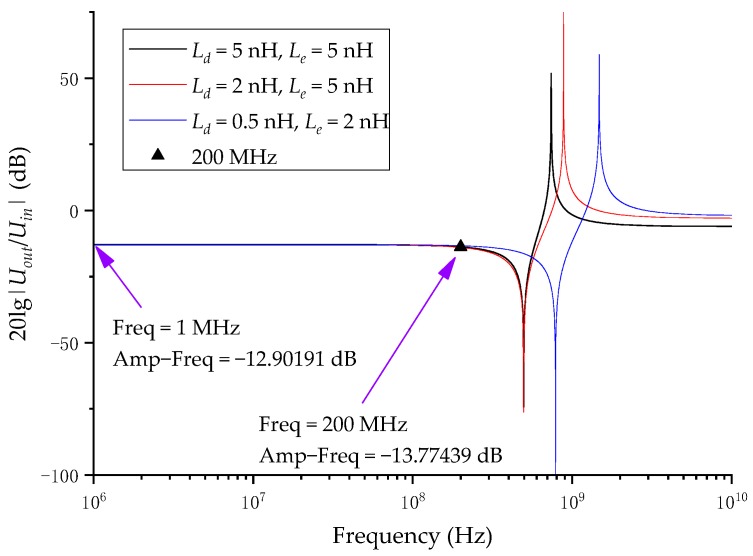
Frequency characteristics affect by inductance under calculated $C_{e}$ and $C_{d}$.

The effect of inductance on the sensor bandwidth was discussed earlier, as shown in Figure 5. In order to verify that the selection of $C_{e}$ and $C_{d}$ will not cause resonance in the measurement frequency band range, the values of $C_{d}$ and $C_{e}$ are brought into Equation (11) and theoretical calculations are performed. Since it is difficult to quantitatively calculate $L_{e}$ and $L_{d}$, in a sensor with similar structure to measure VFTO, this value is an empirical value. In the literature [12,21], the authors have estimated $L_{e}$ and $L_{d}$. These literatures are internal sensors installed at the hand hole of GIS, and their induction plates are larger in area than the sensors at the front of the measurement system in this paper. Therefore, the inductance is relatively large. Since $L_{e}$ is greater than $L_{d}$, $L_{e}$ is mainly estimated. In the literature [12], the estimated $L_{e}$ is about nH level or even smaller, and in the literature [21], the estimated $L_{e}$ is 4–5 nH. Substitute the estimated maximum value of 5 nH in literature [21] and calculate it using Matlab R2019a. The result is shown in Figure 9. In Figure 9, the black triangle is the effect of the inductance on the frequency characteristics at 200 MHz when $L_{e}$ and $L_{d}$ are both 5 nH. The difference between the frequency characteristics at 200 MHz and the frequency characteristics at 1 MHz is within 1 dB, and as $L_{d}$ and $L_{e}$ decrease, the resonance point moves to higher frequencies. In this paper, the area of the induction plate was much smaller than the internal sensors in the literature, and the structure of the induction plate was designed to reduce the inductance, so the inductance was much smaller than the internal sensors. As a result, the frequency response affected by the inductance at 200 MHz could be ignored, so the calculated system parameters would not be affected by inductance, and would not cause resonance at a frequency below 200 MHz.

To verify that the parameter selection meets the needs of the VFTO measurement, we synthesized a simple VFTO signal generated by a single breakdown as defined by IEC 60071-1 to test the model. In the literature [24], the method of synthesizing the VFTO waveform has been studied in detail, in accordance with the requirements of this test. Its frequency components, duration and expressions are as shown in the Table 1.

**Figure 10:**
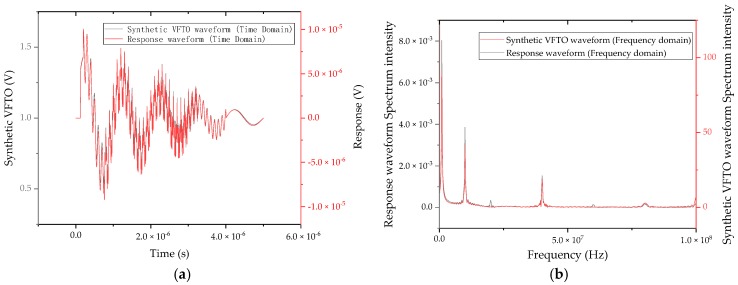
VFTO synthetic waveform excitation test results. (**a**) Time domain spectrum and (**b**) frequency domain spectrum.

The synthesized VFTO waveform is input as an excitation to the model, and the resulting response is shown in Figure 10a, and Fourier transform was performed on the excitation waveform and the response waveform, and the obtained frequency domain response is shown in Figure 10b. The output signal of the time domain range measurement system was consistent with the waveform of the original signal and the time point at which the maximum amplitude occurred. There was no obvious phase shift between the high frequency superposition section of 0.2–4 μs and the low frequency range of 4–5 μs, and the response time to the step voltage at 0.125 μs was also within 3 ns. The high frequency part can quickly follow the changing trend of the VFTO synthesized signal without obvious overshoot and oscillation. In the frequency domain spectrum obtained by simulation, each component in the synthesized VFTO frequency domain spectrum had an obvious response. However, the response obtained at 100 MHz simulation was slightly smaller than the response of the synthesized VFTO signal. However, the overall flatness was within 3 dB, which is an allowable error.

**Figure 11:**
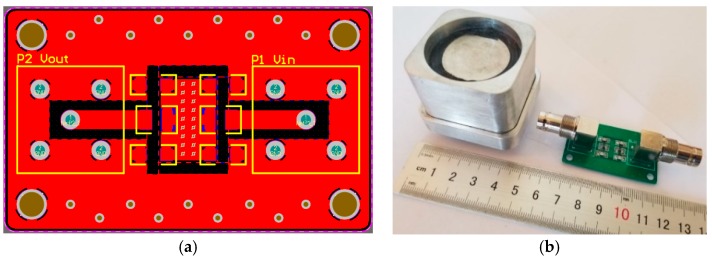
Complete measurement system. (**a**) Differentiating–integrating circuit PCB layout and (**b**) manufactured prototype.

After the parameters are determined, the physical object need to be fabricated, but the theoretical calculation parameters are simplified, and there are many uncertain factors in the actual fabrication, such as the stray inductance of the resistor, the withstand voltage value of the capacitor, the parasitic capacitance and inductance caused by the unreasonable wiring, etc. Although its value is small, it will have a large impact in high frequency sensitive circuits. Therefore, in the differentiating–integrating circuit, it is necessary to select the resistor having a high withstand voltage and a small stray capacitance, also, the capacitor should have a high withstand voltage. In order to reduce the parasitic capacitance and inductance caused by the circuit wiring, the resistor can be arranged in series using multiple identical resistance values, and the capacitor can be radially arranged using multiple identical capacitance values. The result is a reduced area between the components, increasing the distance between the components, and effectively reducing parasitic capacitance and inductance. In terms of component connection selection, in order to avoid parasitic capacitance caused by large via holes of the plug-in components, patch components were used. The final design of the differentiating–integrating printed circuit board (PCB) is shown in Figure 11a, the manufactured prototype measurement system is shown in Figure 11b.

## 3. Measurement System Calibration

Due to machining accuracy and other uncertainties, the manufactured measurement system needs to be calibrated. The measurement system will be time-domain and frequency-domain calibrated separately, and compared with the existing internal VFTO sensor to determine the real performance of the measurement system. In order to avoid the influence of the length of the coaxial cable on the calibration results, the following tests use coaxial cables of the same length and wave impedance.

### 3.1. Frequency Domain Calibration

**Figure 12:**
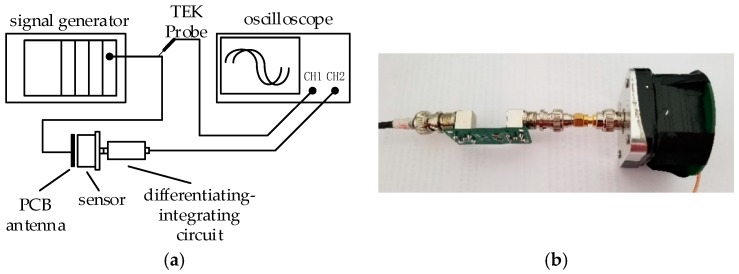
Measurement system sweep frequency experiment. (**a**) Layout and (**b**) experimental setup.

Sweeping frequency to calibration the measurement system and draw a frequency response curve to visually observe the linearity of the sensor’s frequency characteristic. The experiment used the PCB antenna to simulate the limit case, that is, the $C_{d}$ was the largest when the distance between GIS HV busbar and the sensor was zero, and the upper limit bandwidth of the measurement system was the lowest value. The experimental layout is shown in Figure 12.

The signal generator uses a programmable function generator consisting of NI PXIe-1071 and NI PXI-5422 with a maximum output amplitude of 12 V and a frequency output range of 0.000037–80 MHz. The analog electrode uses a PCB antenna and connected to the signal generator through an IPX interface with a small parasitic capacitance. The analog electrode is directly opposite to the sensor and is fixed by a tape, and the analog electrode and the induction plate form a high voltage capacitor. For comparison with the original signal amplitude, the Tektronix P6139B probe was used to connect directly to the signal source, it had a voltage division ratio of 10:1 and a bandwidth of 500 MHz, which is fully compliant with measurement needs. Connect the probe and measurement system outputs to CH1 and CH2 of the oscilloscope Tektronix MSO4104B, observe the output signals of both.

**Figure 13:**
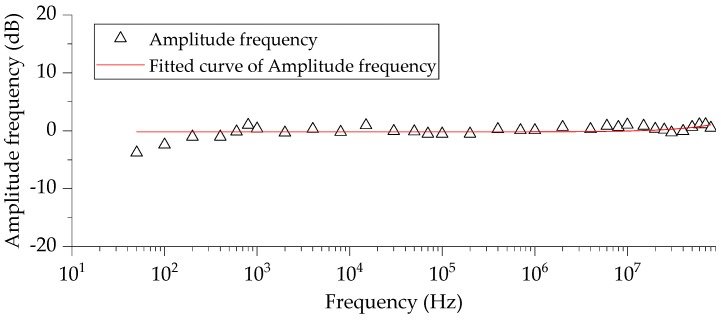
Amplitude–frequency response characteristics of the measurement system.

The response curve measured in the frequency response is shown in Figure 13, the vertical axis is the normalized value of the output voltage and the input voltage, and the horizontal axis is the frequency in Hz. The standardized method uses the Z-score normalization method, and its formula is as follows

(16) $$x^{\prime }=\frac{\left(x-\mu \right)}{\sigma },$$

where *x* is raw data, *μ* is the mean of all data and *σ* is the variance.

It can be seen from the experimental results of Figure 13 that the frequency response of the measurement system was straight, and the voltage division ratio was relatively stable within the test range. Since the maximum frequency of the signal generator was 80 MHz, and the curve did not drop significantly at 80 MHz, the bandwidth of the measurement system was greater than 80 MHz. In addition, linearity of the sensor as within ±1 dB in the range of 0.00005–80 MHz, which means the measurement system has a good amplitude-frequency response in the low frequency range.

**Figure 14:**
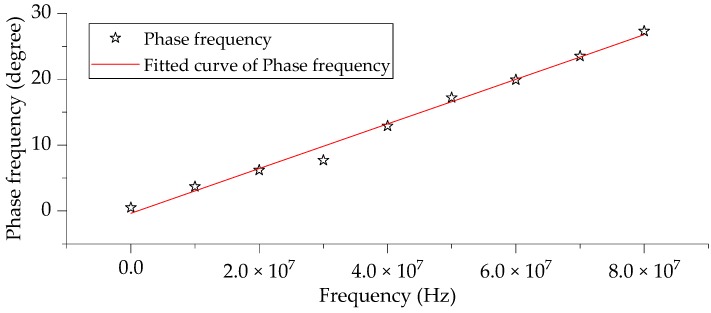
Phase-frequency response characteristics of the measurement system.

Figure 14 shows the phase-frequency response of the measurement system. The experimental data in the figure was fitted to a linear equation with a coefficient of determination reached 0.99072. It can be considered that the phase-frequency characteristic of the measurement system is linear.

### 3.2. Time Domain Square Wave Response Calibration

**Figure 15:**
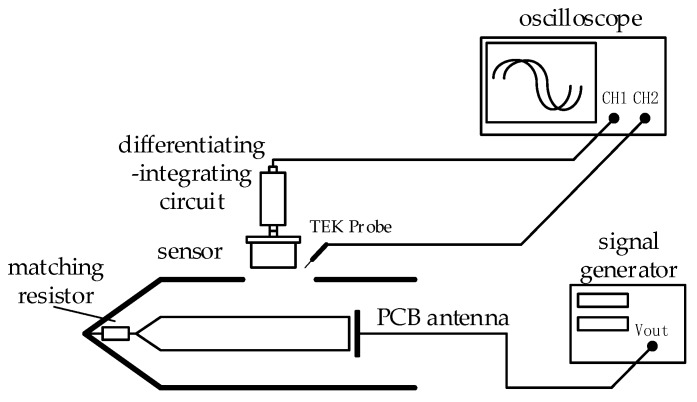
Measurement system square wave response experiment layout.

In order to accurately measure the square wave response of the measurement system, an experimental platform as shown in Figure 15 was built. In the experimental platform, the actual size of the tank and HV busbar of GIS [25] was scaled down. The square wave source uses a self-made steep frontier pulse generator with a maximum amplitude of 500 V and a fall time of 3 ns. Connect the square wave source output to the PCB antenna, and place the PCB antenna inside the tank, 1 cm away from one end of the busbar, to simulate the actual situation when VFTO is generated. The measurement system was placed above the tank observation port, and the Tektronix P6139B probe was placed at the same height as the sensor for comparison. In order to study the influence of the front-end matching and back-end matching of the calculus circuit on the measurement system, two settings were tested.

The scaled reduction model of GIS can be simplified to the coaxial transmission model. The main mode of transmission was the TEM mode, but when the frequency was too high, high-order modes, like TE mode and TM mode, were generated. Both the TE mode and the TM mode have electric or magnetic field components in the direction of waveform propagation, and the size and direction of the opening port will affect the internal electromagnetic field. In all higher order modes, the lowest cutoff frequency is the ${\mathrm{TE}}_{11}$ wave, and the cutoff frequency is

(17) $$\left(f_{c}\right){\mathrm{TE}}_{11}=\frac{c}{\pi \left(a+b\right)},$$

where *c* is the speed of light, *a* is the diameter of the coaxial inner busbar, and *b* is the diameter of the coaxial outer shield. In the model we built, a=0.06 m, b=0.26 m, the cutoff frequency of the ${\mathrm{TE}}_{11}$ wave was 298 MHz, and the high frequency cutoff frequency of the square wave generator was

(18) $$f_{BW}=\frac{0.35}{t_{r}}=116.6\;\mathrm{MHz}\ll \left(f_{c}\right){\mathrm{TE}}_{11}.$$

Therefore, it could be considered that the high-order mode TE mode and the TM mode were not generated in this model, also, opening the observation port according to the transmission direction of the waveform did not affect the internal electromagnetic field.

**Figure 16:**
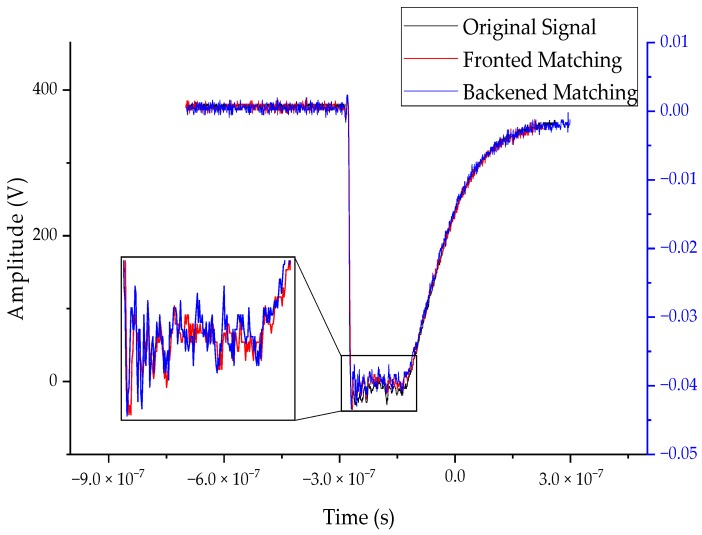
Square wave response curve of the measurement system.

The time-domain characteristic of the square wave response of the sensor measured by the square wave response experiment is shown in Figure 16. In the figure, the horizontal axis is time, the left vertical axis is the output voltage measured by the probe, and the voltages of the two integrators of the sensor are basically the same, so only one coordinate axis of the right vertical axis was set. It can be seen from the square wave experiment result that the waveform measured by the front-end matching and the back-end matching of the integrating circuit was basically the same as the waveform measured by the Tektronix probe. The fall time measured by the probe was 3.08 ns, and the fall time of the front-end matching and the back-end matching was 3.9 ns and 3.4 ns, which were within 1 ns compared with Tektronix probe, and both front-end matching and the back-end matching were less than 5 ns, which met the VFTO measurement requirements. Although the fall time of the back-end matching was less than the front-end matching, the back-end matching waveform oscillates more than the front-end matching, the reason is that the oscillating circuit was in series with the oscilloscope resistor, and the wave impedance of the cable and circuit was different, so the front-end matching was selected.

### 3.3. Voltage Division Ratio Calibration

Voltage division ratio is the characteristic of the measurement system, and the calibration is affected by multiple factors. The circuit breaker and the isolating switch operate at high voltage, which produces pre-breakdown and restrike, causing electromagnetic waves propagate in the GIS tank. Due to different voltage levels, the outer diameter of the GIS tank and the inner diameter of the HV busbar were different, and the amplitude of the electromagnetic wave when it reached the sensor was also different, as a result, there were some differences in the voltage division ratio of the measurement system in GIS equipment with different voltage levels. Therefore, this paper developed a power frequency calibration platform to calibrate the measurement system.

**Figure 17:**
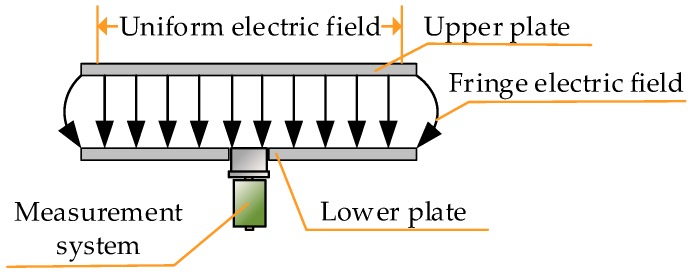
Calibration platform structure.

The calibration platform consists of a calibration voltage source and a test platform, and test platform consists of two circular planar electrodes. The radius of the upper and lower plates was 1 m. The upper plate was connected to the calibration source, and the calibration voltage source generated a high-voltage power frequency signal. The center of the lower plate had a circular opening for the sensor and the other parts were grounded. In addition, the area of the measurement system to be calibrated was negligible relative to the electrode plate of the calibration platform. Therefore, a uniformly distributed time-varying electric field was formed between the two plates of the calibration platform. Distance between the upper and lower plates was set by referring to the distance from the HV busbar to the outer conductor in the 500 kV GIS equipment in Table 2 [25], which was 20 cm. Simulation results show that the measurement system to be calibrated was around by the uniform electric field and not affected by the fringe electric field on the edge of the plates. The structure of calibration platform is shown in Figure 17.

**Figure 18:**
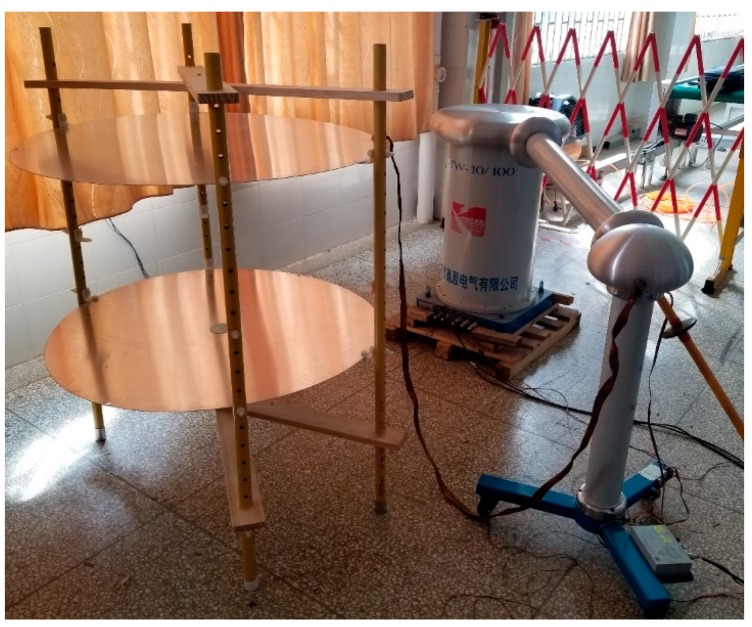
Power frequency voltage division ratio calibration platform.

The calibration voltage source uses YDTW-10/100 non-partial-discharge power frequency test transformer, which consists of control panel, anti-corona test transformer, protection resistor $R_{p}$ and anti-corona coupling capacitor $C_{c}$. It can provide 0.001–100 kV alternating current (AC) output without partial discharge (PD), which can affect the calibration process [26,27,28]. After the test voltage is output from the anti-corona test transformer, it reaches the metal disk through the protective resistor, and the metal disk is connected to the coupling capacitor. The protection resistor and the coupling capacitor form a first-order RC low-pass filter, which can filter the high-frequency signal generated by the transformer, and output to the calibration platform without PD. The calibration system is shown in Figure 18.

**Figure 19:**
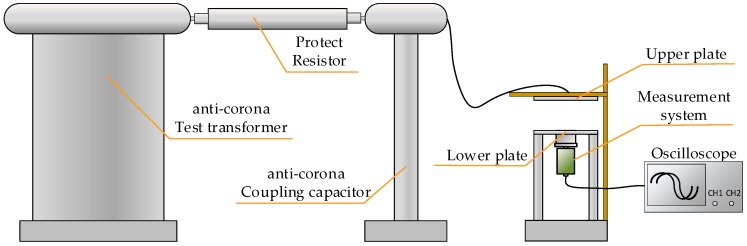
Layout of the voltage division ratio experiment.

**Figure 20:**
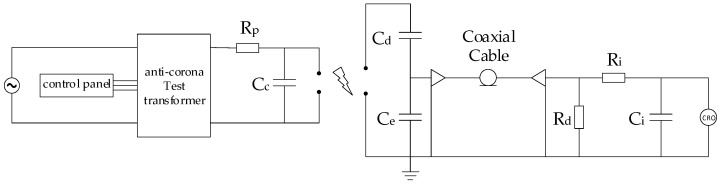
Equivalent circuit of the voltage division ratio experiment.

Place the front sensor of the measurement system into the circular opening of the lower plate, and connect the back end to the oscilloscope to observe the peak value of the waveform. The power frequency excitation of 10–70 kV is injected into the platform using calibration voltage source. Field test arrangement and circuit diagram are shown in Figure 19 and Figure 20.

Table 3 shows the experimental results. It can be seen from Table 3 that the voltage division ratio of the measurement system was basically the same under the power frequency voltage of 10–70 kV, the average value was about 49,881, and the maximum change rate was 2%. Although the high-low frequency voltage division ratio of the measurement system was not completely consistent, but the sweep calibration indicates that the linearity of the sensing system was good, so the voltage division ratio of the measurement system under the high-voltage power frequency calibration could be used as a reference.

### 3.4. Compare with Internal Sensors

The internal sensor was installed at the GIS flange, disassembly and assembly needs to release SF6 gas and refill SF6 gas for GIS, which will affect the stability and insulation performance of GIS. However, because the internal sensor has good high and low frequency performance and a fast response, it can be used to compare the performance of the measurement system developed in this paper. Frequency domain response and time domain response of the measurement system were calibrated in the laboratory environment, the purpose of this experiment was to verify the difference between the output waveform of the measurement system and the existing internal sensor in the actual GIS.

The experiment was carried out on the sensor test platform of the China Southern Power Grid High Voltage Experimental Base. A GIS model of the internal VFTO sensor was used to verify the performance of the measurement system.

**Figure 21:**
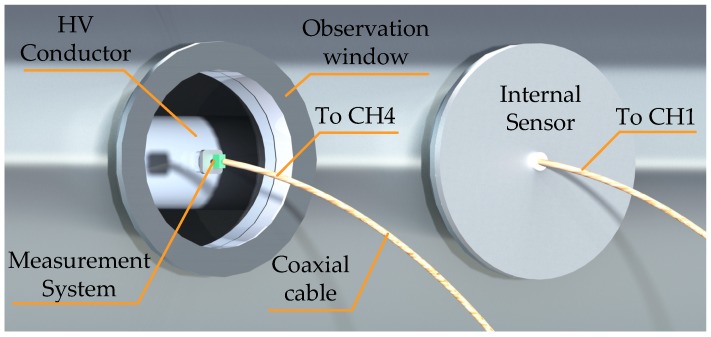
Layout of comparative experiment.

The test layout is shown in Figure 21. Factory preset VFTO sensor was installed at the insulation flange on one side of the GIS model. Meanwhile, sensor of the measurement system developed by this paper was installed at the transparent observation window, 50 cm away from the insulating flange. However, the distance from the HV busbar to the internal sensor was less than the distance from the HV busbar to the sensor of the measurement system in this experiment. The internal VFTO sensor had a nominal bandwidth of 0.00005–100 MHz and a voltage division ratio of 10,000. We used a lightning signal generator with a maximum amplitude of 500 kV as the signal source, connect directly to the signal input at one end of the HV busbar. Connect the internal sensor and measurement system of this article to channel 1 and channel 4 of the oscilloscope Tektronix DPO7254C.

**Figure 22:**
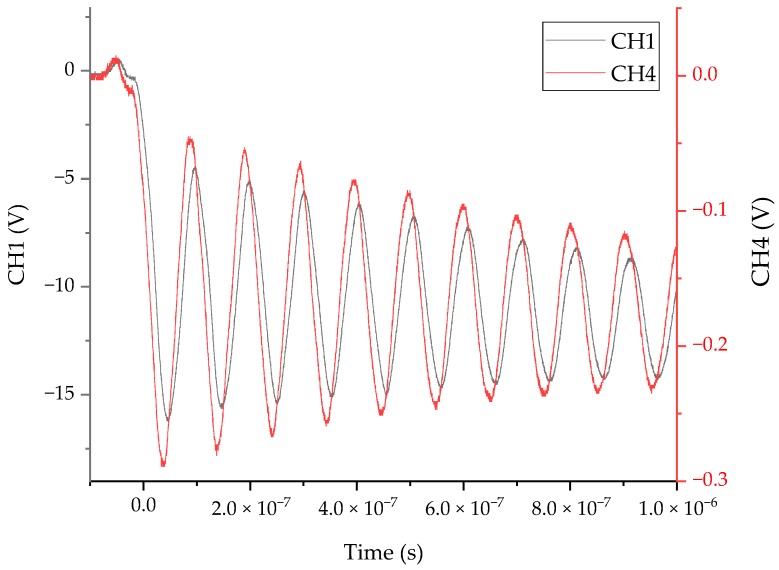
Results of comparative experiment.

Inject 100 kV lightning impulse into the HV busbar and observe the response of the two sensors. The test results are shown in Figure 22. It can be seen from the figure that the waveform frequency of channel 1 and channel 4 was the same. The waveform of the measurement system and the internal VFTO sensor maintains a stable phase shift, indicating that the measurement system had stable followability compared with the internal sensor, and the voltage division ratio was much larger than the internal sensor.

There are two reasons for the phase shift: First, the phase shift generated by the measurement system and second, the distance between the two sensors sensing substrate and the busbar is different, and the electromagnetic wave arrival time is different to produce a phase difference. It can be seen from the phase-frequency calibration experiment that the measurement system would produce a linear phase shift, and the internal sensor was different from the busbar distance of the sensor measurement plate of the measurement system in this paper. Therefore, the stable phase shift was the result of superimposing the above two factors, and did not affect the comparison of the sensor and the internal sensor in this experiment.

## 4. VFTO Measurement in 500-kV GIS

**Figure 23:**
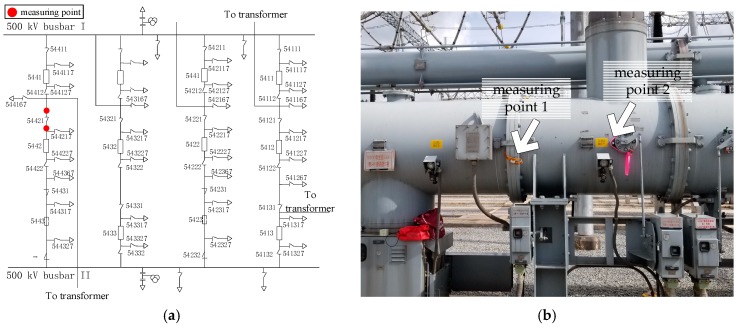
Field measurement. (**a**) Main circuit of DALI substation and (**b**) measuring point.

**Figure 24:**
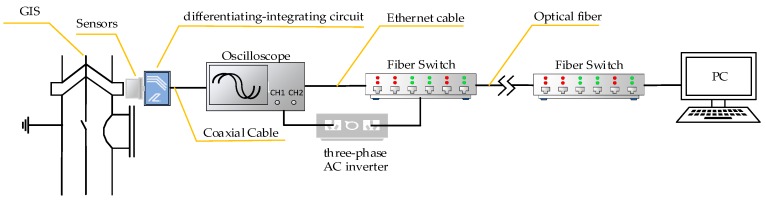
Layout of the field test measurement system.

In order to verify the performance of the measurement system developed in this paper, the measurement system was used to measure the VFTO generated by the isolation switch closing of the 500 kV Dali substation. The main circuit and measuring point distribution of the tested circuit are shown in Figure 23a, and the field measuring point photo is shown in Figure 23b, those two measuring points were respectively located near the isolating switch 54,421, one near the busbar side and the other near the circuit breaker side. Sensors were fixed on the surface of the insulated basin by using tape, followed by a differentiating–integrating circuit, and connected to the oscilloscope through a coaxial cable. The oscilloscope was Tektronix DPO7254C, with a sampling rate of 250 MS/s and 20 M points of storage depth. The oscilloscope was connected to the fiber switch through the Ethernet cable. After the fiber switch converted the electrical signal into an optical signal, the remote computer controlled the oscilloscope through the optical fiber. The layout of field test measurement system is shown in Figure 24.

**Figure 25:**
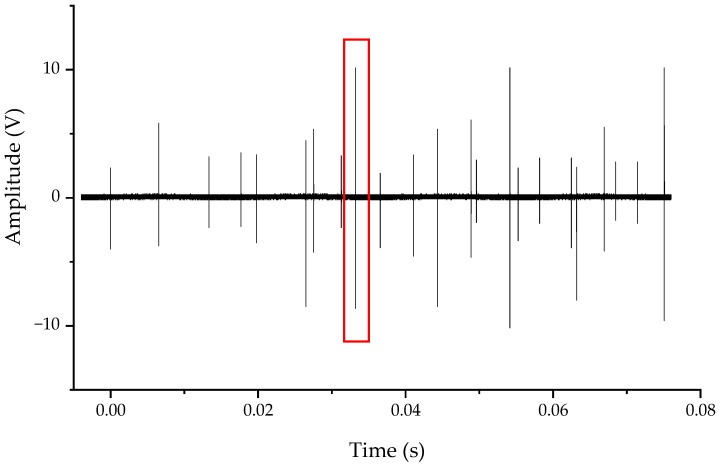
Typical closing waveform measured at point 1, the red box is a typical VFTO shock.

**Figure 26:**
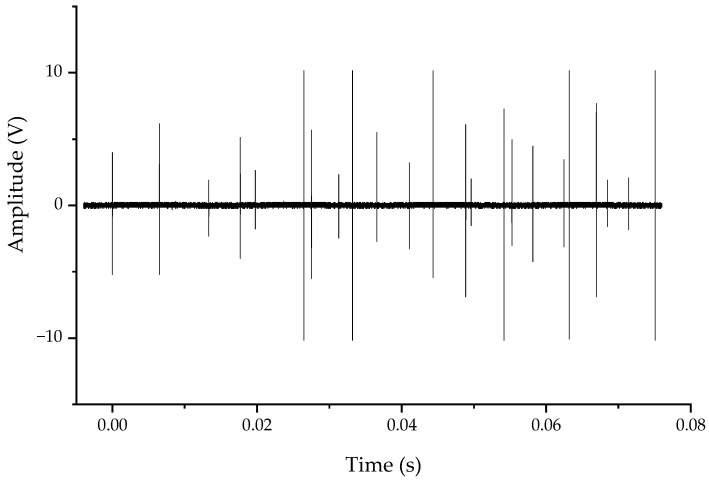
Typical closing waveform measured at point 2.

When the isolating switch was closed, the voltage waveforms of measuring point 1 and measuring point 2 are as shown in Figure 25 and Figure 26. There were multiple spikes during the recording time. These spikes were the VFTO generated by the heavy breakdown of the contact during the closing process of the isolating switch. During the closing process, the distance between the contacts was changed from large to small, and the VFTO oscillating envelope was also changed from large to small. The 550 kV rated voltage isolating switch took 3–4 s to complete the opening and closing action, but the recording time set by the oscilloscope was small, the full waveform of VFTO was not recorded, so the VFTO shock envelope had no obvious small trend.

**Figure 27:**
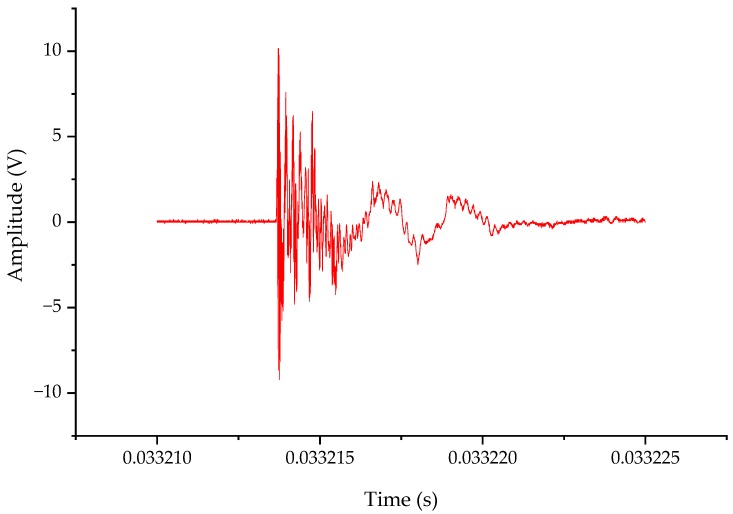
Typical VFTO shock.

**Figure 28:**
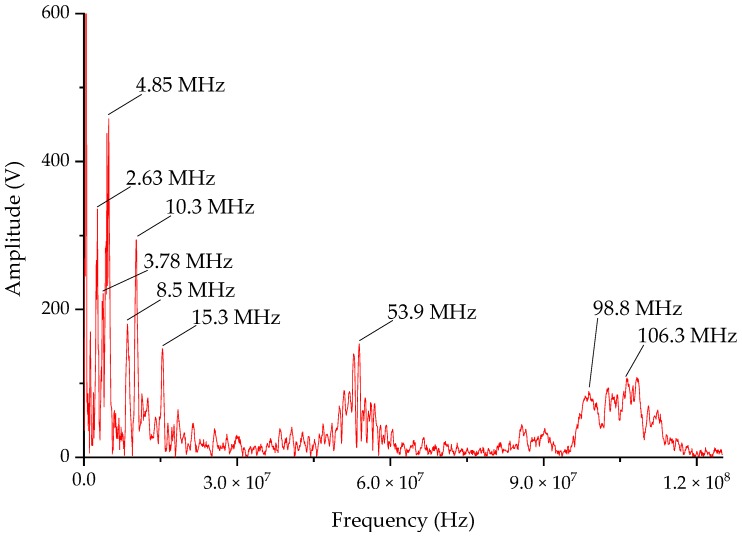
Typical frequency domain spectrum of VFTO.

The spikes in Figure 27 are zoomed in to obtain a single typical VFTO waveform as shown in Figure 25. It can be seen from the time domain waveform of Figure 27 that the continuous oscillation time was about 6 μs, and there was a step voltage. High frequency part was mainly concentrated in the first 2 μs, and the peak value of the VFTO was 1.22 p.u. ($500\;\times \;\sqrt{2}/\sqrt{3}$ was the base unit) [29]. An FFT analysis was performed on this waveform to obtain a result shown in Figure 28. It can be seen from Figure 28 that the measured VFTO frequency was mainly concentrated at 0.1–5 MHz, 10 MHz, 50 MHz and 100 MHz, which conformed to the definition of VFTO waveform in IEC 60071-1.

According to the measured results, the measurement system developed by this paper could successfully detect the VFTO. The time domain curve and the frequency domain curve were in accordance with the classical theory of VFTO and the experimental data support measured by other methods. The VFTO waveform under this experimental setup could be measured accurately.

## 5. Conclusions

This paper focused on the design and optimization of the VFTO measurement system. The measurement system consisted of a front-end capacitive divider and a back-end differentiating–integrating circuit. It satisfied both compact size and high performance based on accurate measurement of VFTO. A frequency-domain, time-domain, voltage divide calibration experiment and comparison experiment with an internal VFTO sensor was performed, a field experiments to verify the actual performance of the measurement system was also conducted. The result of this paper could be summarized as follows:

(1) In order to improve the performance of the measurement system, the front-end capacitive divider used a special structural design to reduce stray inductance at high frequencies, and the back-end differentiating–integrating circuit used patch component and radial PCB components to reduce circuit parasitic capacitance;
(2) An experimental platform was built, the measurement system was calibrated by frequency swap and high-voltage square wave using a self-made pulse source. The calibration results and the measured results show that the voltage division of the sensor from 50 Hz to more than 100 MHz was relatively stable, and the square wave response time was less than 1 ns from the measured fall time from the Tektronix probe;
(3) A power frequency and voltage division ratio calibration platform were developed, which consisted of a calibration voltage source and a test platform. The process is guaranteed to be unaffected by the fringe electric field when calibrating voltage division ratio. The power frequency voltage divider of the measurement system was relatively stable, and the average value was 49981:1;
(4) The field measurement was carried out in the Dali 500 kV substation. In the limit isolation switch measurement, the measured VFTO frequency was mainly distributed between 0.1 and 100 MHz, 0.1–5 MHz, 10 MHz, 50 MHz and 100 MHz was more obvious.

## Figures and Tables

**Table 1:** Frequency component and duration of VFTO synthesized signal.

Frequency Component/MHz	Equations	Time/μs
0.00005	cos(100πt)	0–5
1	$0.5e^{-5\times 10^{5}t}\sin\left(2\;\times \;10^{6}\pi t\right)$	0.125–5
10	$0.2e^{-3\times 10^{5}t}\cos\left(2\;\times \;10^{7}\pi t\right)$	0.2–4
40	$0.1e^{-10^{5}t}\sin\left(8\;\times \;10^{7}\pi t\right)$	0.75–3
80	$0.05\cos\;\left(1.6\;\times \;10^{8}\pi t\right)$	1.75–2.25, 2.3–2.8
100	$0.05\cos\;\left(2\;\times \;10^{8}\pi t\right)$	0.125–0.625, 2.5–3.25

**Table 2:** GIS structure size for different voltage levels.

Voltage Levels	110 kV	220 kV	500 kV	1000 kV
Outer conductor diameter D/mm	240	360	520	890
HV busbar diameter d/mm	60	90	120	260

**Table 3:** Power frequency divider ratio of measurement system under different voltage levels.

Calibration Voltage/kV	Response Peak/V	Measurement System Voltage Division Ratio
10	0.202	49,504.95
20	0.402	49,751.24
30	0.594	50,505.05
40	0.806	49,627.79
50	0.992	50,403.23
60	1.198	50,083.47
70	1.420	49,295.77
Average		49,881.64
